# Construction of an American mink Bacterial Artificial Chromosome (BAC) library and sequencing candidate genes important for the fur industry

**DOI:** 10.1186/1471-2164-12-354

**Published:** 2011-07-08

**Authors:** Razvan Anistoroaei, Boudewijn ten Hallers, Michael Nefedov, Knud Christensen, Pieter de Jong

**Affiliations:** 1 University of Copenhagen, The Faculty of Life Sciences, Department of Basic Animal and Veterinary Sciences, Division of Animal Genetics and Bioinformatics, Groennegaardsvej 3, Frederiksberg C, Denmark; 2 Children's Hospital Oakland Research Institute, BACPAC Resources, 747 52nd Street, Oakland, California 94609-1809, USA

## Abstract

**Background:**

Bacterial artificial chromosome (BAC) libraries continue to be invaluable tools for the genomic analysis of complex organisms. Complemented by the newly and fast growing deep sequencing technologies, they provide an excellent source of information in genomics projects.

**Results:**

Here, we report the construction and characterization of the CHORI-231 BAC library constructed from a Danish-farmed, male American mink (*Neovison vison*). The library contains approximately 165,888 clones with an average insert size of 170 kb, representing approximately 10-fold coverage. High-density filters, each consisting of 18,432 clones spotted in duplicate, have been produced for hybridization screening and are publicly available. Overgo probes derived from expressed sequence tags (ESTs), representing 21 candidate genes for traits important for the mink industry, were used to screen the BAC library. These included candidate genes for coat coloring, hair growth and length, coarseness, and some receptors potentially involved in viral diseases in mink. The extensive screening yielded positive results for 19 of these genes. Thirty-five clones corresponding to 19 genes were sequenced using 454 Roche, and large contigs (184 kb in average) were assembled. Knowing the complete sequences of these candidate genes will enable confirmation of the association with a phenotype and the finding of causative mutations for the targeted phenotypes.

Additionally, 1577 BAC clones were end sequenced; 2505 BAC end sequences (80% of BACs) were obtained. An excess of 2 Mb has been analyzed, thus giving a snapshot of the mink genome.

**Conclusions:**

The availability of the CHORI-321 American mink BAC library will aid in identification of genes and genomic regions of interest. We have demonstrated how the library can be used to identify specific genes of interest, develop genetic markers, and for BAC end sequencing and deep sequencing of selected clones. To our knowledge, this is the first report of 454 sequencing of selected BAC clones in mammals and re-assures the suitability of this technique for obtaining the sequence information of genes of interest in small genomics projects. The BAC end sequences described in this paper have been deposited in the GenBank data library [HN339419-HN341884, HN604664-HN604702]. The 454 produced contigs derived from selected clones are deposited with reference numbers [GenBank: JF288166-JF288183 &JF310744].

## Background

The American mink (*Neovison vison*, formerly *Mustela vison*) is a member of the *Mustelidae *family in the order *Carnivora*, an order that includes hundreds of widely distributed wild species as well as common companion animals. Mink have been farmed since the mid-19th century in North America and the early 20th century in Europe. The mink industry has recorded a gradual increase in production with almost 51 million mink pelts harvested globally in 2010 (Finnish Fur Sales [FFS] & Kopenhagen Fur Report, 2010).

As farming of mink is growing, the need to identify the genomic mechanisms for specific traits is becoming more important for breeding, management, and health care of this species. A large Quantitative Trait Loci (QTL) project for mink, comprising more than 1000 F_2 _animals scored for more than 50 traits, has recently been run as a collaborative venture between the Faculty of Agricultural Sciences of the University of Aarhus and the Department of Basic Animal Sciences of the University of Copenhagen, Denmark. In conjunction with the currently existing linkage maps [1,2], our BAC resource now provides a valuable tool for the mapping and characterization of traits involved in production. To identify genomic regions responsible for specific traits, with the ultimate goal of implementation into breeding and management programs, genomic large-insert libraries have been previously proven to be of crucial importance. Large-insert BAC libraries can be screened using gene or genetic markers to identify and map regions of interest. Furthermore, large-scale mapping can utilize libraries in genome projects, and hence provide valuable data on the genome structure. To date, the focus of mink research has been on coat color genetics [3-9], isolating microsatellite markers [10,11], developing linkage maps [1,2], gene and comparative mapping using Zoo-FISH experiments [12,13], and somatic cell hybrids [14-16].

In the last 15 years, BAC libraries have been extensively used in physical mapping and complete eukaryote genome sequencing [17]. The utility of BAC clones as substrates for end sequencing, in conjunction with advanced DNA techniques and microarray analysis, has permitted construction of robust physical maps and selection of BAC minimum tiling paths. Recent advances in deep sequencing technologies (454 Roche pyrosequencing, Illumina sequencing, etc.) have created powerful opportunities in which BAC libraries play an important role, as this study demonstrates. Additionally, BAC end sequences (BESs) not only provide a snapshot of the sequence composition of the genome of the species of interest [18] but also aid in genome assembly [19], chromosome walking [20], creating comparative physical maps [21], and identifying genetic markers [22].

Here, we present the availability and utility of an American mink BAC library. This is the first reported *Neovison vison *BAC library; it will be an important tool for constructing physical maps and for the identification and sequencing of regions of the mink genome. As the present paper proves, these large-insert BAC clones are useful for identification of regions of interest to the fur industry as well as to the fundamental science community. The quantitative characteristics, which are most often a common breeding objective, shall also be considered at the genetic level. Coat color genetics in mink is the first interest targeted, as variation is common; the fur color, markings (if any), or the patterns separate the color types. It is established that there are at least 31 different genes that control color types in the standard mink, counting both recessive and dominant ones [5]. This study is aimed at candidate genes for the most popular colors as well as some other traits, as presented in Table 1. It is also the first reported study of mammals in which BAC clone availability in conjunction with new sequencing technologies have produced complex information in a small genome project.

**Table 1:** Candidate genes for which CHORI-231 was screened and subsequently 454 sequenced

Candidate gene probe	Probe sequence source	No. of positive signals as evaluated by no. of T7 BACs hits	Phenotype(s)/condition potentially involving the candidate genes	**Acc. no**.	Coverage of the gene 454 sequenced	Content of the clone(s) being sequenced	SSRs in the clone(s) contig	No. of contigs per gene	No. of overlaping clones included for 454 sequencing	Size of genomic information generated (in Kb)
***KIT ligand *(*KITL*)**	Dog and human	4	*Roan, Spotted, and White phenotypes (excluding Albino but including Hedlund white*, associated with *deafness)*	JF288175	Complete	*KITL*	13	83	2	226
***Microphthalmia-associated transcription factor *(*MITF*)**	Dog	12		JF288172	Complete	*MITF*	23	46	2	205
***KIT *(*CD117 *or *C-KIT*)**	Mink & dog	4		JF288168	Clone missing exon 1	*KIT*	26		2	160
***Melanophilin *(*MLPH*)**	Dog	11	*Silver*	JF288169	Complete	+*COL4A1*	18	38	2	267
***Lysosomal trafficking regulator *(*LYST)***	Dog	4	*Alutian *color (associated with Chediak-Higashi syndrome)	JF288176	Complete	*LYST*	11	42	2	274
***Silver *(*SILV or PMEL*)**	Dog	1	*Blue/silver *phenotypes	JF288177	Complete	Gene rich	27	1	2	183
***Tyrosinase *(*TYR*)**	Mink	12	*Albino *and *Himalayan *type	JF288171	Complete	+ *GRM5, NOX4 *&*RPL23A*	38	32	2	320
***Protein atonal homolog 1 *(*Atoh-1*)**	Dog	10	*Hedlund white*, associated with *deafness*	JF288173	Complete	*Atoh-1*	23	30	3	195
***Melanocortin 2/3 Receptor *(*MC2R ***&***MC3R*)**	Dog	6	Involved in a wide range of physiological functions, including pigmentation	JF288181	Complete	+ *MLC5R, KPNA2 *&*RNMT*	5	13	3	97
***Fibroblast growth factor 5 ***(***FGF5*)**	Dog	5	Hair length	JF288167	Complete	*+ LDHR *&*PRDM8*	22	33	*2*	177
***R-spondin-2 ***(***RSPO2*)**	Dog	7	Hair growth and coarseness	JF288170	Clone missing exon 5	*RSPO-2*	30	24	1	153
***Melanocyte stimulating hormone *(*POMC*)**	Dog & human	7	Various types of pigmentation	JF288182	Missing 15 nt in exon 2 at 5'	*+ EFR3 & DNMT3A*	17	79	*2*	223
***Melanocortin 1 Receptor *(*MC1R*)**	Mink & dog	1	*Palomino, Pastel, Pearl & Reddish *phenotypes	JF288183	Complete	Gene rich	2	96	1	124
***Solute carrier family 24, member *(*SLC24A5*)**	Dog	5	Differences in skin pigmentation	JF310744	Missing 9 nt in exon 1 at 5'	*CTXN2 *&*MYEX2*	17	37	*2*	218
***Agouti related protein *(*AGRP*)**	Dog	5	*Agouti *and *Pearl *phenotypes loci were found to be closely linked	JF288166	Missing 106 nt in exon 2 at 5'	Gene rich	10	20	1	193
***Integrin-B *(*ITGB1*)**	Dog	6	*Aleutian Disease Virus *(ADV) and *Influenza *susceptibility/resistance	JF288179	Missing over 500 nt in several exons	*ITGB1*	15	70	1	105
***Major Histocompatibility Complex, class II, DR beta 1 *(*HLA-DRB1***)	Dog	3		JF288174	Missing 160nt in one exon	*RPL7A*	19	33	1	139
***B-defensine *(*DEFB1*)**	Dog	4		JF288178	Inconsistent cds		5	51	2	186
***Keratin71 *(*KRT71*)**	Dog	15	Coarseness	JF288180	9 exons covered	10 members of the *KRT *family	3	101	2	56
***Transmembrane inner ear *(*TMIE*)**	Dog	None	*Hedlund white *associated with *deafness*	-	-	-	-	-	-	-
***Tyrosinase-related protein 1 *(*TYRP1*)**	Dog	None	Associated with pigmentation genetics	-	-	-	-	-	-	-

## Results and discussion

### 1. Library characterization

Based on analysis of *Not*I digested DNA isolated from 131 clones, the average insert size of the CHORI-231 BAC library [23] was estimated to be 170 kb with approximately 3% false positive (noninsert) clones. With a total of approximately 166,000 clones and a mean insert size of 170 kb, the mink BAC library collectively contains 28,220 Mb of mink DNA. The size of the mink genome is unknown. However, the haploid DNA content of the domestic ferret *Mustela putorius furo*, the closest relative to the mink among species studies, is 2.81 pg [24], i.e., its genome size is approximately 2700 Mb. Assuming that the genome size of the American mink is similar to that of the ferret (i.e., 2700 Mb), our BAC library affords roughly 10 genome equivalent (10X) of the mink genome (i.e., 28220 Mb/2700 Mb = 10.45).

### 2. End sequencing of BAC Clones. Comparative mapping of mink BESs to the human and dog genomes. Mink genome characterization

A total of 2505 high-quality BESs were obtained from sequencing both ends of 4 randomly chosen 384-well plates of American mink BAC clones, as well as from sequencing the T7 ends of the selected 220 clones that had been screened for genes of interest. Only BESs that were at least 200 bp long were used in the statistical and sequence composition analyses. The combined length of sequence analyzed was in excess of 2 Mb, and included 866 paired-end BESs (sequence available for both ends of a BAC clone). The average length of individual BESs was 862 bp. BESs were deposited in GenBank [GenBank: HN339419-HN341884, HN604664-HN604702].

Considering the high degree of synteny between human and mink [12], the existing Zoo-FISH data involving the dog, mink, and human [13], and the relative accuracy of the reference human and dog genomes sequences, we BLASTed the mink BESs to the human and dog genomes (BUILD 37.1 and 2.1, respectively). Of the total of 2505 high-quality BESs, 177 (7%) BESs gave unique hits (at a cutoff value of e-10) to the human genome and a total of 266 (10.6%) to the dog genome. The density of the mink BESs on the human genome is rather sparse (also due to the rarity of coding sequence), but owing to the stringent cutoff used for the comparative mapping analysis it is more accurate. The comparative BLASTing against the dog and human genomes revealed distances between the mink insert ends of 133 kb and 184 kb, respectively. This observation supports the previous synteny data determined by Zoo-FISH in which the number of rearrengments between dog and mink is much greater than that between human and mink [12,13].

Overall, the BESs had an average GC content of 41.3%, which is similar to the 41% GC content of the human genome [25]. An internal search for the repetitive elements on BESs revealed 17 different types of repeats of which 14 were carnivore specific while only 3 (17%) were "*Mustelidae *family" specific when searched against the public database. The representation of the "*Mustelidae*" specific repeats account for roughly 2% of the analysed sequences. No American mink specific type of repeat was detected. A carnivore RepeatMasker analysis on the BESs revealed that 25% of the total sequence consisted of transposable elements (TEs), 5.5% of which were SINEs and 16.5% were LINE elements (ratio LINE/SINE of 3:1). Even when adding the 2% "Mustelidae" specific elements, the proportion of repeat sequences in the mink BESs is suggestively different from that found in the dog genome at 34% [26]. This implies that the mink genome may be smaller than the canine counterpart. The virtual, comparative map of the mink genome provides the foundation from which to construct a mapping tool for the identification of genes underlying economically important traits.

### 3. Microsatellite analysis

A search for simple sequence repeats (SSRs) in the mink BES dataset revealed 131 repeat sequences (Table 1) found in 119 BESs (0.5% of the total BESs). The most frequently occurring SSRs were dimer (34%) and tetramer (27%), followed by monomer repeats (25%). Pentamer, trimer, and hexamer repeats were present at much lower frequencies, accounting for only 14% of the microsatellites present. The microsatellite occurrence rate in the mink genome seems to be approximately one every 15 kb. Additionally, each assembled contig containing genes had a variable number of SSRs (Table 1), which subsequently could be developed into microsatellite markers.

### 4. Transcribed regions

After masking for TEs, a MEGABLAST (dbEST downloaded from NCBI) comparison revealed that 122 of the mink BESs (0.7%) were similar to human proteins at an E value of <e-10. An accurate estimate for the total length of the protein coding fraction in the mink genome does not currently exist. Nevertheless, this small resource adds additional information to the 1558 existing mink cDNA sequences deposited at GenBank [GenBank: ES609118-ES610847] (Anistoroaei & Christensen, unpublished data).

### 5. Screening of the library

The CHORI 231 BAC library was screened using 39 probes specific to known expressed sequences representing 21 candidate genes potentially involved in color phenotypes as well as candidates for traits involving fur coarseness, hair length, and health-related conditions in mink (Table 1). Most of them were created from dog and 2 from mink EST sequences (Table 1) using the "Universal Probes" tool set for carnivores [27]. These probes were hybridized to the BAC filters as a single pool, and 220 BAC clones were verified as positive for 19 different expression tags after T7 end sequencing and comparison of the sequence to the dog assembly (Table 1). Although we cannot accurately determine the number of positives for each individual gene as some of the BESs did not provide information in relation to any gene, based on the number of clones and the observed average insert size of 170 kb, we estimate the library to have an approximately 10-fold genome representation.

To identify the relationships between the probes and the clones, BAC T7 end sequencing was performed for the arrayed positive clones and BLASTed against the dog and human genomes. Nineteen of the 21 genes taken into consideration (*KIT, KITL, MLPH, LYST, TYRP1, MC1R, TYR, PMEL, DEFB1, ITGB1, HLA-DRB1, DFNA17, TMIE, AGRP, MITF, MSH, SLC24A5, MC2/3R, RSPO2, FGF5*, and *KRT71*) were identified by comparing the T7 BACs to the dog genome sequences (BUILD 2.1).

### 6. 454 sequencing of the clones containing genes of interest

As described in the "Methods" section, two rounds of BAC clones organized in pools were sequenced independently in Germany ("*Germany pool*") and California ("*California pool*") by two different approaches. The obtained information varied to some extent between the two pools. Thus, the *Germany pool *had fewer gaps (from no gaps in *SILV *assembled clones to 82 gaps in *KITL *assembled clones) in the sequences (Table 1) and the sum of the contigs from clones for individual genes (one single or two overlaping clones per locus) averaged approximately 240 kb. Longer parts of the clone(s) were sequenced and the total read data summed up to 16 Mb. Statistically, this allocates approximately 2.6 Mb of sequence per gene (2 clones each) representing 10- to 20-fold coverage.

The *California pool *yielded a shorter average insert size and there were more gaps in the contig (up to 100, as in *KRT71*) (Table 1) with the sum of the contigs from clones for individual genes (one single, two or three overlapping clones per locus) averaging approximately 155 kb. The total read data summed up to 33 Mb, which translates into 10- to 20-fold coverage. In this case, some sequences were found to match outside of the expected syntenic region, probably due to the inconsistencies in the dog genome assembly. The generated sequence for each locus is presented in Table 1.

**Figure 1:**
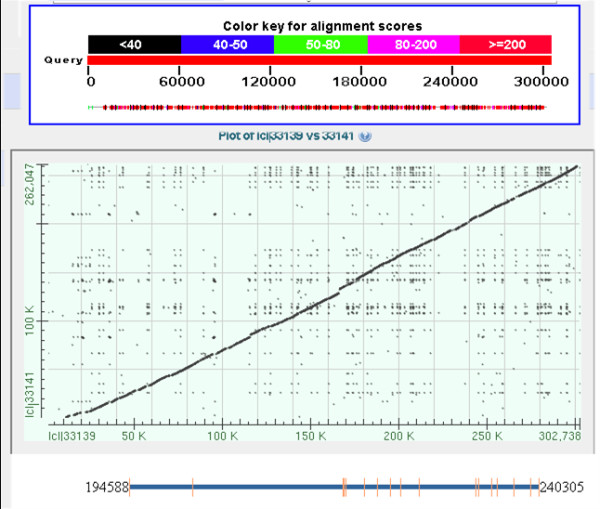
**Linearity between dog (X axis) and mink (Y axis) for more than 250 kb of assembled sequence, which contains the MLPH gene**. The small black dots represent repeat sequences in both dog and mink.

The maximum contig spanned 80,852 bp, but a few of the clones had contigs shorter than 5000 bp. The quality of the 454 sequencing could be evaluated, as 4 of the genes had been sequenced both in the *Germany *and *California *pools and from different clones (Table 2). The results indicate that in the *MLPH *case it is the same allele that has been sequenced, whereas the other 3 genes have a much higher error rate, indicating that 2 different alleles have been sequenced. Many of the gaps coincided with single base repeats, which is a known problem with the 454 sequencing system. The error rate might be slightly higher, as when the BLAST program finds too many mismatches it can cut the query sequence into 2 pieces. The general assembled contigs were subsequently aligned using the dog assembly as a reference and, in most cases, the linearity of the sequence is consistent (Figure 1). Exon/intron boundaries for each of the genes have been established using "gene finding" tools [28,29]. The analysis indicates that, in most cases, the coding sequences are entirely embedded in the contigs of the genes. Additionally, sequences from 3 different clones could be assembled and aligned providing the information for the entire LINE element in the American mink (GenBank: JF288184).

**Table 2:** Accuracy of the 454 sequencing shown by comparisons of the 4 genes that have been sequenced in 2 different batches (represented by different clones)

Gene	Base pairs overlap	Base differences	Gaps	"Error rate"	BLASTs	Comments
*MLPH*	132,208	19	65	0.063%	20	Same allele
*KITL*	81,545	63	146	0.256%	36	Different allele
*SILV*	75,069	70	174	0.325%	19	Different allele
*KIT*	35,344	23	99	0.345%	22	Different allele

For *MLPH*, the assumption is that the same allele was sequenced, while for the other 3 genes, different alleles were compared

## Conclusions

Providing a publicly available redundant genomic large-insert library for the American mink was important for several reasons. First, large genomic insert clones can be used to construct contigs for regions of interest, can be fingerprinted on a large scale and thus used to create large physical maps of the mink genome, or can be used for shotgun sequencing approaches. Second, the exchange of data between researchers is improved when they are utilizing the same library. The characterization reported here illustrates the usefulness of the library for identifying genomic clones and the possibility of utilizing BAC clones in gene mining projects. The large average insert size of the clones combined with the high redundancy will provide researchers with the possibility of obtaining complete gene sequences within a single BAC clone. This might be useful for expression studies of genes and their regulatory elements. Third, the BAC library of the American mink will be an important tool for future mustelides projects and to the fur industry. This information can be used to improve breeding and management programs, leading to increased profitability for the industry through the provision of basic data that will be usable in schemes for selecting desirable traits. The utility of the library also resides in the possibility of targeted sequencing of the gene-specific, selected clones by means of the new deep sequencing technology. This has already been implemented for 19 genes selected from this library and, when completed, the results will be communicated in later reports.

To our knowledge this is the first targeted 454 sequencing from clones containing genes of interest in mammals and proves to be accurate and useful in the context of a small animal genetics project. Considering the high degree of synteny between the existing Zoo-FISH data derived from the dog, mink, and human [13] and the relative accuracy and linearity of the reference dog genome sequences [2], the mink BACs were BLASTed against the dog genome; the dog is the closest relative to the mink and therefore its genome was utilized in the assembly of the clones (Figure 1). The dog genome assembly can be used as a reference in relation to the mink sequence but caveats apply as there are some inconsistencies due either to the old Zoo-FISH inconsistencies for small segments of the genome or to the errors in the dog genome assembly [2].

We have constructed a high-quality 10 × BAC library for *Neovison vison *and demonstrated the utility of the library as a genetic tool. Further screenings of the library with other genes of interest involving traits important for the fur industry are under way. This will facilitate further research in the field of skin and fur physiology and function.

## Methods

### 1. High molecular weight DNA preparation

The spleen of a 6-month old male American mink with the *Wild *type color from the *Faculty of Life Sciences Experimental Mink Farm, Taastrup, Denmark *was frozen immediately after harvesting. High-molecular weight DNA was prepared and embedded in InCert agarose plugs according to the standard procedures [30] for frozen tissue.

### 2. Insert preparation

The InCert agarose-embedded, high-molecular weight DNA was partially digested with a combination of 5 U *Eco*RI and 100 U *Eco*RI methylase. Double size fractionation of partially digested DNA was done on a CHEF apparatus (BioRad). After selecting the desired size fractions, agarose noodles representing the various fragment sizes above 150 kb were electrodialyzed (by unidirectional electrophoresis) in dialysis membranes for DNA concentration and recovery.

### 3. BAC vector preparation

The pTARBAC2.1 BAC vector was digested with *Eco*RI, treated with calf intestine phosphatase (Roche), and separated on 1.0% agarose gel. The vector fragment was purified from the gel as previously described [31].

### 4. Construction of the BAC library

The BAC library was constructed following the standard protocols [30-32] using the pTARBAC2.1 vector [33]. The ligation products were transformed into electrocompetent *E. coli *DH10B T1 phage-resistant cells (Invitrogen). High-density replica filters were prepared.

### 5. Insert size analysis

To determine mink BAC library insert size, 131 randomly selected BAC clones were digested with *Not*I and analyzed by PFGE.

In addition, approximate insert sizes were estimated by comparative blasting of 2499 BAC-end sequences against dog and human assemblies (BUILD 2.1 and 37.1, respectively).

### 6. Hybridization screening

A set of 39 36-mer overgo probes from unique genomic sequences were radioactively labeled using P32 and were hybridized to the set of 9 filters. All probes used were derived from potential candidate genes for coat colors and traits important for the mink farming and fur industry, as shown in Table 1. Hybridization was carried out at 65°C, overnight, in Church buffer [34]. The filters were washed 4 times at 65°C, each for 15 minutes, using 1.5 × SSC and 0.1% SDS. Positive signals were evaluated by exposing the filters to Phosphor Image cassettes (Amersham Biosciences). All of the clones identified in the screening were re-arrayed into new 96-well plates in preparation for end sequencing.

### 7. BAC end sequencing

BAC end sequencing was performed by the Genome Center at Washington University, St. Louis, Missouri 63108, USA. Sequencing reactions were performed using BigDye™ Terminator cycle sequencing chemistry and the following primers: T7: 5'- and KBR/TJ.

### 8. End sequences analysis

#### Repeats Discovery

The program used to identify microsatellite sequences within BESs consisted of a custom-made PERL script, developed at the University of Copenhagen, that identified sequences containing mono-, di-, tri-, tetra-, penta-, and hexa-nucleotide repeats.

#### Repetitive Elements

Repeat analysis was conducted using the web-based program RepeatMasker [35] with carnivore selected as the DNA source as well as an ab initio repeat identification program derived from RepeatScout [36].

#### Gene Ontology

After the BESs were masked for repeats, the BLASTX function was used to screen for protein coding regions. The non-redundant protein sequence database was used for the analysis, with a cutoff value of e-10. Only matches to human proteins in the database were recorded.

#### Comparative Mapping of Mink BESs to the Human and Dog Genomes

The BESs were then blasted against the human and dog respectively reference genomes (NCBI build 36.3 and build 2.1) not including alternate assemblies, [37] to estimate the overall distribution of the random clones within the genome and only unique hits were considered. A unique hit is defined as a match at a cutoff value of e-10.

### 9. 454 GSX sequencing of the selected BAC clones

#### Sequencing

Sequencing was performed in 2 distinct rounds, using 2 different approaches. In the first round (named *Germany pool*), clones representing 6 different genes (*c-KIT, KITL, MLPH, LYST, TYR*, and *PMEL) *were individually prepared and bar coded. One-eighth of a 454 picotitroplate (approximately 35 Mb of sequence) was used for the sequencing. The second round (named *California pool*) contained DNA prepared as a pool from clones individually grown clones representing 19 genes (*c-KIT, KITL, MLPH, MC1R, PMEL, DEFB1, ITGB1, HLA-DRB1, DFNA17, TMIE, AGRP, MITF, MSH, SLC24A5, MC2R, MC3R, RSPO2, FGF5*, and *KRT71*) and subsequently run together (no bar coding) on one-fourth of a 454 picotitroplate. Four of the genes were sequenced in both pools (Table 2).

#### Analysis of the Assembled Contigs

Assembled contigs were BLASTed against the dog genome assembly and analyzed. Long transposable elements were also analyzed to evaluate the accuracy of the 454 sequencing in this context.

## Authors' contributions

RA and BTH performed the experiments. RA drafted the manuscript. MN provided supervision. KC provided the sequence analysis and sequence data interpretation. PDJ and RA coordinated the project. All authors read and approved the final manuscript.
